# Origin and Epidemiological History of HIV-1 CRF14_BG

**DOI:** 10.1371/journal.pone.0024130

**Published:** 2011-09-28

**Authors:** Inês Bártolo, Ana B. Abecasis, Pedro Borrego, Helena Barroso, Francine McCutchan, Perpétua Gomes, Ricardo Camacho, Nuno Taveira

**Affiliations:** 1 Unidade dos Retrovírus e Infecções Associadas, Centro de Patogénese Molecular, Faculdade de Farmácia de Lisboa, Lisboa, Portugal; 2 Centro de Investigação Interdisciplinar Egas Moniz (CiiEM), Instituto Superior de Ciências da Saúde Egas Moniz, Caparica, Portugal; 3 Centro de Malária e Outras Doenças Tropicais, Instituto de Higiene e Medicina Tropical, Lisboa, Portugal; 4 Bill and Melinda Gates Foundation, Seattle, Washington, United States of America; 5 Laboratório de Biologia Molecular, Centro Hospitalar Lisboa Ocidental, Hospital Egas Moniz, Lisboa, Portugal; Institute of Infectious Disease and Molecular Medicine, South Africa

## Abstract

**Background:**

CRF14_BG isolates, originally found in Spain, are characterized by CXCR4 tropism and rapid disease progression. This study aimed to identify the origin of CRF14_BG and reconstruct its epidemiological history based on new isolates from Portugal.

**Methodology/Principal Findings:**

C2V3C3 *env* gene sequences were obtained from 62 samples collected in 1993–1998 from Portuguese HIV-1 patients. Full-length genomic sequences were obtained from three patients. Viral subtypes, diversity, divergence rate and positive selection were investigated by phylogenetic analysis. The molecular structure of the genomes was determined by bootscanning. A relaxed molecular clock model was used to date the origin of CRF14_BG. Geno2pheno was used to predict viral tropism. Subtype B was the most prevalent subtype (45 sequences; 73%) followed by CRF14_BG (8; 13%), G (4; 6%), F1 (2; 3%), C (2; 3%) and CRF02_AG (1; 2%). Three CRF14_BG sequences were derived from 1993 samples. Near full-length genomic sequences were strongly related to the CRF14_BG isolates from Spain. Genetic diversity of the Portuguese isolates was significantly higher than the Spanish isolates (0.044 vs 0.014, P<0.0001). The mean date of origin of the CRF14_BG cluster was estimated to be 1992 (range, 1989 and 1996) based on the subtype G genomic region and 1989 (range, 1984–1993) based on the subtype B genomic region. Most CRF14_BG strains (78.9%) were predicted to be CXCR4. Finally, up to five amino acids were under selective pressure in subtype B V3 loop whereas only one was found in the CRF14_BG cluster.

**Conclusions:**

CRF14_BG emerged in Portugal in the early 1990 s soon after the beginning of the HIV-1 epidemics, spread to Spain in late 1990 s as a consequence of IVDUs migration and then to the rest of Europe. CXCR4 tropism is a general characteristic of this CRF that may have been selected for by escape from neutralizing antibody response.

## Introduction

By the end of 2009, the estimated number of adults and children living with HIV/AIDS in Portugal was 42,000 (32,000–53,000) [1]. The HIV/AIDS prevalence was 0.6% (0.4%–0.7%) in the adult population, one of the highest in Western Europe [1]. After an initial period dominated by homosexual transmission of HIV-1, a shift towards transmission through heterosexual contacts and drug injection occurred and, today, heterosexual contact is the main route of HIV-1 transmission in Portugal [2]. African and Brazilian immigrants contribute substantially for the number of AIDS cases in this category [2].

The current HIV-1 epidemic in Portugal is caused by multiple subtypes, with predominance of subtype B (41.7%) followed by G (29.4%) [3], [4]. The high prevalence of these two subtypes has promoted the appearance of different types of B/G recombinant strains [4], [5], [6], [7], [8], [9]. CRF14_BG was the first epidemic CRF composed of subtypes B and G to be characterized by full-genome sequencing. This CRF was first isolated in 2002 from intravenous drug users (IVDUs) in Galiza, Spain [10]. CRF14_BG displays a mosaic structure with two inter-subtype breakpoints delimiting a B subtype segment comprising most of gp120 and the 5′ half of gp41, whereas all remaining regions are classified as subtype G [10]. So far, only seven CRF14_BG isolates have been characterized by full-genome sequencing. These were obtained from Spanish (5/7, 71%), Portuguese (1, 14%) and German (1, 14%) IVDUs patients [10], [11]. Until 2007, several sub-genomic sequences related to CRF14_BG were reported in Germany (1), Italy (2), United Kingdom (2), Estonia (15), Spain (38) and Portugal (50) suggesting that this CRF spread efficiently throughout Europe [4], [6], [7], [8], [11], [12], [13], [14], [15], [16], [17], [18], [19], [20], [21], [22]. However, in recent years very few mentions have been made to this CRF in Europe suggesting that its prevalence has reduced significantly [23]. Striking and unique features of most isolates belonging to this CRF are their CXCR4 tropism and association with rapid CD4+ T cell depletion and disease progression [20], [21], [23], [24].

To better understand the epidemiology of CRF14_BG we have characterized the full-length genome of three new CRF14_BG isolates obtained from three Portuguese patients infected in 1997, dated the origin of this CRF and reconstructed its evolutionary history. Moreover, to trace back the epidemiological history of this virus, *env* gene sequences were obtained from 62 patients infected in Portugal between 1993 and 1998. Finally, to gain some insight into the selective forces promoting CXCR4 usage by isolates belonging to this CRF, we have used genetic methods to determine the tropism of a significant number of recent Portuguese isolates and phylogenetic methods to investigate positive selection in the V3 region. Our results indicate that CRF14_BG originated in Portugal in the beginning of the HIV-1 epidemics. From here, it probably spread to Galiza, Spain, in late 1990 s and to other countries in Europe in early 2000. Our results confirm that the CXCR4 tropism is a general and stable feature of CRF14_BG and suggest that this phenotype might be a consequence of successful escape from neutralizing antibody response.

## Results

### Molecular epidemiology of partial and near full-length HIV-1 sequences

**Figure 1:**
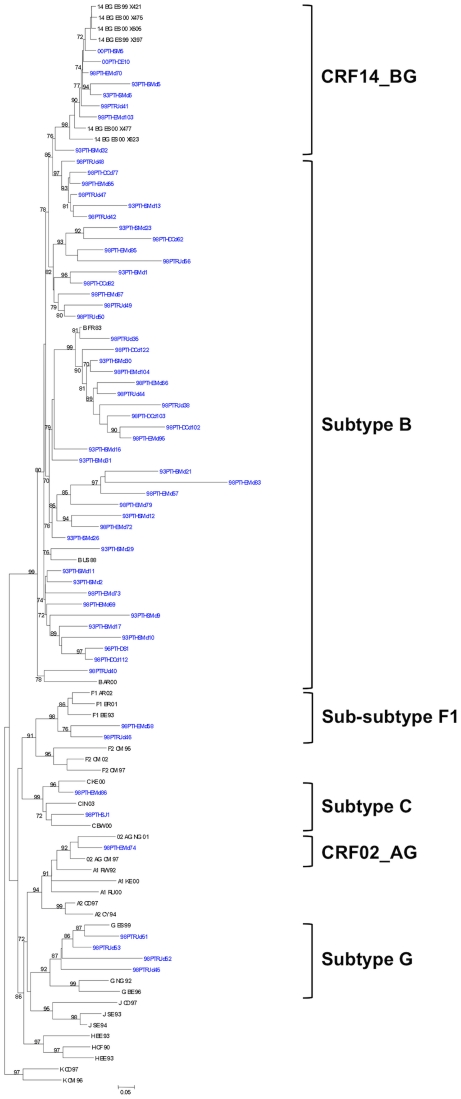
Phylogenetic analysis of Env gene sequences from HIV-1 infected patients. The maximum likelihood phylogenetic trees were constructed with reference sequences from all HIV-1 subtypes. The bootstrap values supporting the internal branches defining a subtype or a CRF are shown. Bootstrap values of 70% or greater provide reasonable confidence for assignment of an individual segment to one or the other genotype. The scale represents number of base substitutions per site.

Phylogenetic analysis showed that HIV-1 C2-C3 sequences belonged to different subtypes (Figure 1). As expected, subtype B was the most prevalent subtype (45 sequences; 73%) followed by CRF14_BG (8; 13%), G (4; 6%), F1 (2; 3%), C (2; 3%) and CRF02_AG (1; 2%). Importantly, three CRF14_BG sequences were derived from 1993 samples. These results suggest that CRF14_BG was already circulating in Portugal in 1993.

**Figure 2:**
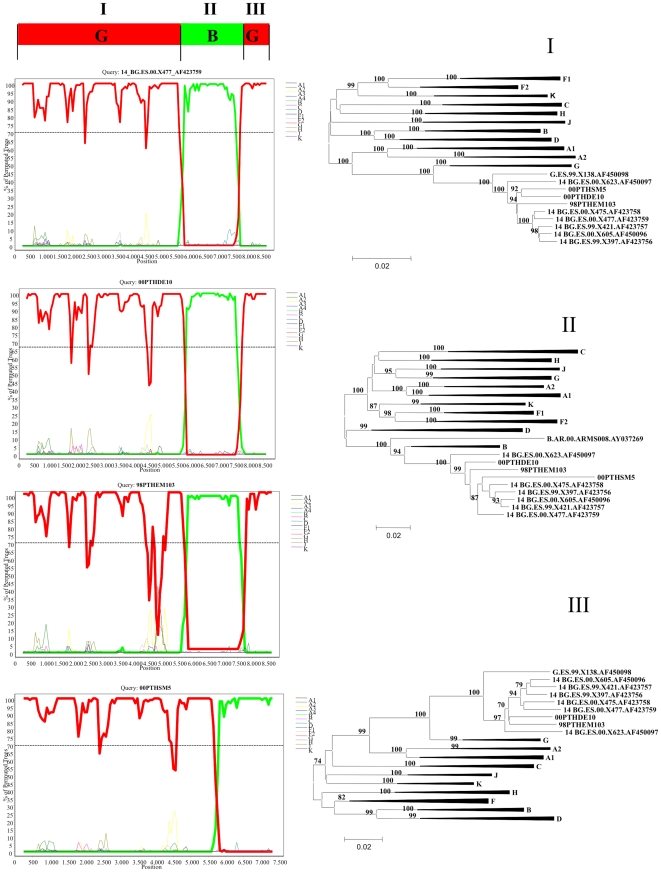
Bootscanning analysis of full-length genomes from Portuguese and Spanish CRF14_BG isolates. The dashed line indicates the cut-off of 70%. The maximum likelihood phylogenetic trees were constructed with reference sequences from all HIV-1 subtypes. The bootstrap values supporting the internal branches defining a subtype or a CRF are shown. Bootstrap values of 70% or greater provide reasonable confidence for assignment of an individual segment to one or the other genotype. The scale represents number of base substitutions per site.

Near full-length genomic sequences were obtained from three HIV-1 infected patients residing in Lisbon. These were two children (00PTHSM5, 00PTHDE10) infected by vertical transmission in 1997 and one young adult (98PTHEM103) infected by heterosexual contact in the same year (Table 1) [24]. Bootscan analyses revealed that the new isolates share a mosaic structure that is similar to the reference CRF14_BG strains with only two intersubtype breakpoints delimiting a B subtype segment comprising most of gp120 and the 5′ half of gp41 and the remaining portions of the genome of subtype G (Figure 2). Phylogenetic analyses revealed that the different sub-genomic sequences were strongly related with reference CRF14_BG isolates from Spain.

**Table 1:** Epidemiological characterization of CRF14_BG infected patients.

Sample	Gender	Ethnic group	Year of infection	Transmission route	GeneBank accession number
00PTHSM5	F	Caucasian	1997	MTCT	GU230138
00PTHDE10	M	Caucasian	1997	MTCT	GU230137
98PTHEM103	M	Caucasian	1997	Heterosexual^a^	GU230139

MTCT - mother to child transmission;

a Individual infected by sexual contact with HIV infected female sex worker which was intravenous drug user [24].

### CRF14_BG originated in Portugal in early 1990 s

**Figure 3:**
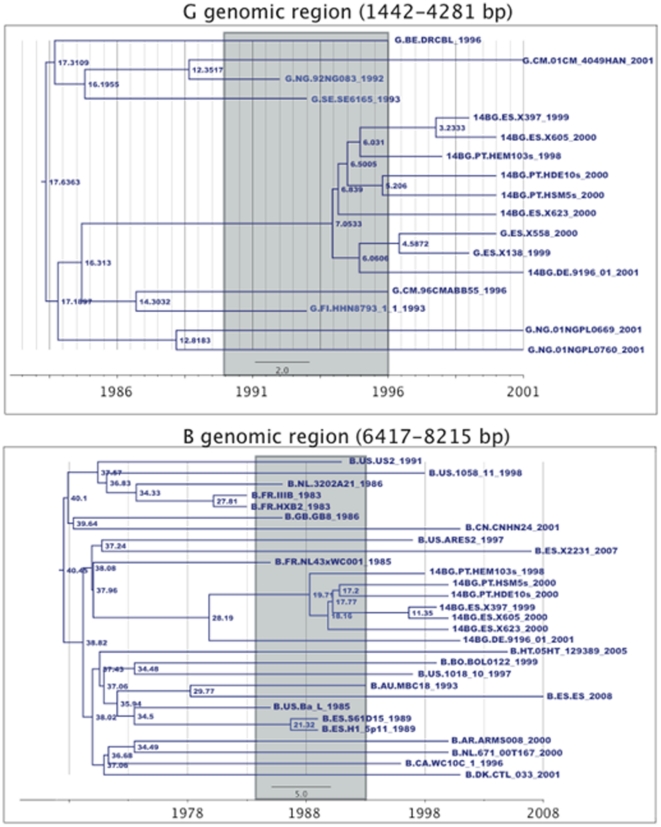
Maximum Clade Credibility Tree for the subtype G and subtype B genomic regions of the CRF14_BG. The 95% confidence interval for the date of origin of CRF14_BG is indicated in both trees as a grey square. Node values indicate its time of origin. Sequence names are coded as [Subtype].[Country].[Seqname]_[Year of Sampling].

The mean date of origin of the CRF14_BG cluster was estimated to be 1992 (range 1989 and 1996) based on the subtype G genomic region and 1989 (1984–1993) based on the subtype B genomic region (Figure 3). The Portuguese CRF14_BG genome sequences were not monophyletic, but clustered with Spanish CRF14_BG sequences. Therefore, no discrimination could be made between the time of entry of this CRF in Portugal and in Spain. Notably, two full-length subtype G sequences from Spain (G.ES.00.X558 and G.ES.99.X138) clustered within the CRF14_BG cluster, indicating a possible subtype G ancestor for this CRF.

On the other hand, despite a similar divergence rate to the MRCA between Portuguese and Spanish isolates (0.030 substitutions per site vs 0.024, P = 0.2857) the genetic diversity between Portuguese isolates was significantly higher than between the Spanish isolates (0.044 vs 0.014, P<0.0001). CRF14_BG isolates from Portugal were found in all transmission groups and some partial env CRF14_BG-like sequences were obtained from samples collected back in 1993 whereas original Spanish CRF14_BG isolates were all obtained in 2000 from IVDUs. These data is consistent with a long standing presence of this CRF in Portugal and suggest that CRF14_BG originated in Portugal rather than in Spain.

### Most CRF14_BG isolates use CXCR4

**Figure 4:**
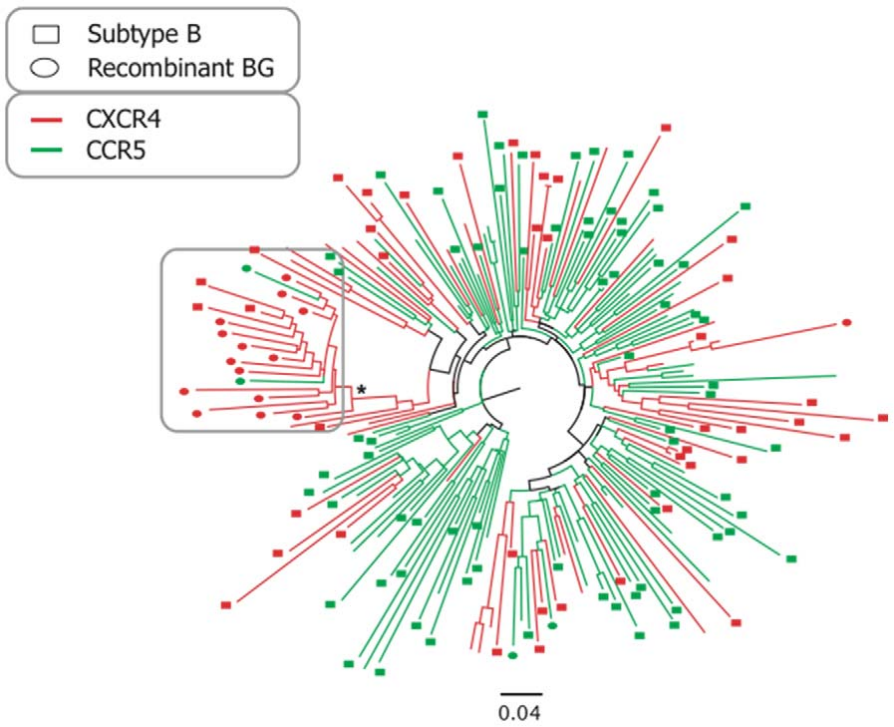
Phylogenetic tree for the C3-V3 genomic region of the recombinant BG sequences, sequences downloaded from the Los Alamos database and from Portuguese patients, showing the significant clustering of BG sequences and associated CXCR4 usage. Sequences are labeled according to subtype and coreceptor usage, Sequences were either subtype G in pol and subtype B in env (circles), subtype B both in pol and in env (squares). If the sequences were subtype B only in env (no information available for pol), no label was added. Sequences colored green use CCR5 only, while sequences colored red can use CXCR4. Asterisk indicates significant support for the cluster (LRT >0.95). Tree was built as described in the methods section.

Geno2pheno predicted that most (15/19; 78.9%) recombinant G*pol*/B*env* sequences (corresponding to the full-genome CRF14_BG sequences recombination pattern) used CXCR4, while only 4 used CCR5. Notably, the phylogenetic tree of the recombinant BG sequences and control subtype B sequences from the Portuguese and Los Alamos database indicated a cluster of CXCR4 using sequences that included 14 of the BG sequences that used CXCR4 together with 3 other subtype B control sequences that also used CXCR4 and only 2 CCR5 using BG recombinants (cluster had 19 sequences, of which 17 used CXCR4, 89.5%, LRT value of the cluster = 0.98) (Figure 4). If we extend the cluster backwards, we find a 25 sequences cluster (subtype B and recombinant BG) of which 23 use CXCR4 (92%, LRT = 0.82). In no other cluster of the tree did we identify such a high proportion of CXCR4 using strains, indicating that there may be something innate in these sequences that make them evolve to using CXCR4 more frequently than other sequences.

### Positive selection might explain the evolution to CXCR4 usage in CRF14_BG isolates

We analyzed selective pressure both in the full tree and in the BG recombinants cluster. The selective pressure analysis of the complete tree consistently identified in the two models positive selection in amino acid 11 of the V3 loop, which is a main determinant of co-receptor usage (Table 2) [25], [26]. Amino acids 20 and 35 were also consistently identified as being positively selected. Furthermore, the SLAC method also indicated amino acids 21 and 23 of V3 as being under positive selective pressure and the dual variable rates model further indicated amino acid 26. When we analyzed only the BGs subtree (19 sequences), we found no evidence of positive selection in V3 when using the SLAC model while when using the dual variable rates model the amino acid 22 was indicated as being positively selected (Table 2).

**Table 2:** Positive selective pressure on the V3 loop of the analysed dataset.

	Codons under selective pressure^1^	
Method	Whole Tree (Bs+BGs) (Number of sequences in cluster = 201)	BGs cluster (Number of sequences in cluster = 19)
SLAC	11, 20, 21, 23 and 35	None
Dual variable rates	11 , 20, 26 and 35	22

1 Codons identified by Hyphy as being significantly (P<0.05) under selective pressure are indicated.

## Discussion

We provide new molecular and epidemiologic evidence suggesting that CRF14_BG emerged in Portugal in the early 1990 s soon after the beginning of the HIV-1 epidemic. This was surely a direct consequence of the early co-circulation of subtypes B and G among the HIV-1 infected population. In fact, we show here that three CRF14_BG-like isolates were already present in Lisbon in 1993. Definitive proof of the early presence of CRF14_BG in Portugal was obtained by genomic sequencing of three isolates obtained from patients infected in 1997 and representing the two most important transmission groups (vertical and heterosexual transmission). Molecular clock analysis indicated that the ancestor of the Portuguese CRF14_BG viruses dates back to the early 90 s. The early presence of CRF14_BG in these transmission groups implies that it was rapidly converted into a highly successful epidemic strain.

CRF14_BG was found in Galiza, Spain, in 2002 among HIV-1 infected IVDU patients of Spanish (5 patients) and Portuguese (1 patient) origin [10]. Between 1999 and 2007 CRF14_BG-like strains were found abundantly in Portugal, Spain and other European countries [3], [4], [6], [8], [11], [12], [13], [14], [15]. In Portugal, in 2003, CRF14_BG prevailed over all other recombinants [6], [8]. Since then, however, CRF14_BG prevalence decreased significantly in Portugal [3], [9] and Spain [23] and, to our knowledge, it has not been reported elsewhere in the world. One reason for this decrease in prevalence of CRF14_BG might be related with its high tendency for recombination with other subtypes or recombinant forms. This is suggested by the multiple CRF14_BG-like sub-genomic fragments that have been described in the recent literature [9], [18] and by the existence of at least three other BG intersubtype CRFs (CRF20_BG, CRF23_BG and CRF24_BG) [27].

Alternatively, CRF14_BG prevalence may have decreased due to its unusually high pathogenicity. We show here that most CRF14_BG isolates circulating in Portugal form a single cluster and use the CXCR4 co-receptor. The majority of CRF14_BG isolates from Spain also use CXCR4, even those obtained from patients at early stages of infection [20], [21], [23]. In subtype B infected subjects, baseline infection with a CXCR4-using virus is strongly associated with a greater decrease in CD4+ T cell count over time and a greater risk of disease progression [28], [29], [30]. Consistent with this, a rapid decrease in CD4+ T cell counts has been observed in all patients infected with CRF14_BG isolates [21]. Moreover, we have shown recently that CRF14_BG infected patients can progress very quickly to AIDS and death [24]. Taken together, these results provide strong argument to suggest that, like HIV-1 subtype D, CRF14_BG may be highly pathogenic [31], [32].

We show that positive selection acts differently in the V3 loop of CRF14_BG isolates compared to B isolates. In fact, between 0–1 amino acids are under selective pressure in CRF14_BG V3 loop whereas in subtype B these are 4–5. Of particular interest in this context was the finding that amino acid 11 in the V3 loop, which is a main determinant of co-receptor usage [25], [26], was not under selective pressure in the CRF14_BG cluster of viruses. These findings suggests that strong conformational and/or functional constrains prevent changes in the V3 loop of this CRF and implies that the CXCR4 tropism is a stable phenotypic feature of CRF14_BG isolates. Neutralizing antibodies are the main selective forces acting on the HIV-1 envelope and escape from these antibodies can promote rapid envelope evolution [33], [34]. CXCR4 tropism has been associated with escape from neutralizing antibody response both in HIV-1 infection and HIV-2 [35], [36], [37]. Hence, CXCR4 tropism in CRF14_BG might have been a direct consequence of successful escape from neutralization in infected subjects. In this context, it is important to note that the only R5 CRF14_BG isolate described so far was found in a individual that progressed to AIDS and death in only 7 months without producing HIV antibodies (seronegative infection) [24].

In conclusion, CRF14_BG probably emerged in Portugal in the early 1990 s soon after the beginning of the HIV-1 epidemics and spread to Galiza, North of Spain, in late 1990 s as a consequence of the mobility of HIV-1 infected IVDUs. Until 2007 CRF14_BG spread efficiently in Europe and elsewhere and from then on there was a significant decrease in its detection. CXCR4 tropism is a unique characteristic of this CRF that may have been selected for by escape from neutralizing antibody response. The reasons for the current low prevalence of this CRF remain unknown but may be related with high recombination rate with other subtypes or recombinant strains and/or with unusually high virulence and pathogenicity.

## Materials and Methods

### Sample collection and sequencing

HIV-1 blood samples were collected from 62 HIV-1 patients infected between 1993 and 1998 in the North (Porto) and South (Lisbon) of Portugal. Viral genomic RNA was extracted from plasma and reverse transcribed. A nested PCR technique was used to amplify a 409 pb HIV-1 C2-C3 *env* region as described elsewhere [38]. PCR products were sequenced using the BigDye Terminator Cycle sequencing kit (Applied Biosystems), and an automated capillary sequencer (ABI PRISM 310, Applied Biosystems). Three patients residing in the Lisbon, two children infected by vertical transmission and one adult infected by heterosexual contact, all infected in 1997, were selected for full-length genomic sequencing (Table 1). For this study, chromosomal DNA was extracted from *post-mortem* tissue (patient 98PTHEM103) or from peripheral blood mononuclear cells (00PTHSM5, 00PTHDE10) using a universal extraction method as described elsewhere [39]. Full-genome PCR amplification and sequencing was done as described elsewhere [40].

### Subtyping of HIV-1 sequences

The genomic sequences were aligned with reference sequences obtained from the Los Alamos HIV Sequence Database using *Clustal-X* [22] and manual adjustments were made using *Genedoc* [41]. To confirm recombination events and identify recombination breakpoints, bootscanning analysis was performed using *Simplot 3.5.1* [42]. Maximum likelihood analyses [43] were performed using the best-fit models of molecular evolution as estimated by *Modeltest* under the Akaike information criterion [44]. These were the TVM model for the full-genome sequences and TVM+G+I for the C2-C3 sequences. Tree searches were conducted in *PAUP v4.0b10* using a nearest-neighbor interchange heuristic search strategy and bootstrap.

### Dating the origin of CRF14_BG

To date the origin of CRF14_BG, two non-recombinant regions of the genome were used (1442–4281 bp relative to HXB2 - subtype G genomic region; 6417–8215 bp relative to HXB2 –subtype B genomic region). Sequences collected from the Los Alamos database (http://www.hiv.lanl.gov/) were aligned to our 3 new full-genome CRF14_BG sequences. The Los Alamos sequences included 4 CRF14_BG sequences from Spain and Denmark (14_BG.DE.01.9196_01, 14_BG.ES.00.X605, 14_BG.ES.00.X605 and 14_BG.ES.99.X397). Furthermore, for the subtype G genomic region, 10 subtype G sequences were included (G.ES.00.X558, G.ES.99.X138, G.BE.96.DRCBL, G.CM.01.01CM_4049HAN, G.CM.96.96CMABB55, G.FI.93.HH8793_1_1, G.NG.92.92NG083, G.NG.x.01NGPL0669, G.NG.x.01NGPL0760 and G.SE.93.SE6165); while for the subtype B genomic region, 22 subtype B sequences were included (B.FR_IIIB_1983, B.US_ARES2_1997, B.US_Ba_L_1985, B.US_US2_1991, B.FR_NL43×WC001_1985, B.AU_MBC18_1993, B.ES_S61D15_1989, B.AR_ARMS008_2000, B.BO_BOL0122_1999, B.CN_CNHN24_2001, B.CA_WC10C_1_1996, B.US_1018_10_1997, B.US_1058_11_1998, B.NL_671_00T167_2000, B.DK_CTL_033_2001, B.ES_X2231_2007, B.HT_05HT_129389_2005, B.ES_ES_2008, B.ES_H1_5p11_1989, B.FR_HXB2_1983, B.GB_GB8_1986 and B.NL_3202A21_1986). The estimation of the date of origin of the tMRCA of CRF14_BG_was performed using BEAST v1.5 [45]. The model of evolution used was HKY85 with a 4 class gamma distribution to model rate variation among sites and allowing for a proportion of invariable sites. A relaxed molecular clock model implemented under a flexible demographic model (Bayesian skyline plot) was used to date the origin of CRF14_BG as described previously [46]. A prior uniform distribution was set for the date of origin of the phylogeny with a uniform interval between 1901 and 1998. Two BGs clusters – one including only Portuguese CRF14_BG sequences and another including both Portuguese and Spanish CRF14_BG sequences – were defined. These were given uniform prior distributions for the root of the clade between 1931 (the date of origin of HIV-1 as published by Korber et al [47]) and 1998 (1998 was the date of sampling of the oldest sampled CRF14_BG sequence, indicating that the recombinant certainly existed in that year).

### Co-receptor usage, selective pressure and divergence rates estimation

Pairwise genetic distances and divergence rates of Portuguese and Spanish CRF14_BG isolates were calculated as described previously [48].

For coreceptor usage analysis, we included new partial *env* sequences in our alignments. The alignments now spanned the C3-V3 region and included 201 sequences. These were sequences collected either from the Los Alamos database or sequences from Portuguese patients collected for the purpose of coreceptor usage determination before starting Maraviroc treatment [9]. If a patient had a subtype G sequence from *pol* and a subtype B sequence from *env*, it was classified as a BG recombinant (circles in Figure 3). If a patient had a subtype B sequence both in *pol* and *env*, it was classified as a pure subtype B (squares in Figure 3). For the patients collected from the Los Alamos database, sometimes there were only subtype B sequences in *env*; these sequences were left unclassified (taxa not marked with circles nor squares in Figure 3). In total, 201 sequences were included in the alignment, of which 19 corresponded to BG recombinants, 114 were pure subtype B (subtype B in *pol* and *env*) and the remaining were Los Alamos subtype B sequences only in *env* (subtype B in *env*, but unknown subtype for *pol*).

Co-receptor usage prediction of each sequence was made using the *geno2pheno* software [49], after codon-aligning the C3-V3 genomic region with the *GeneCutter* tool available at the Los Alamos website (http://www.hiv.lanl.gov/content/sequence/GENE_CUTTER/cutter.html).

The estimation of the underlying phylogenies for the estimation of selective pressure was made with the *PhyML* software, using the HKY85 substitution model with gamma distributed rate variation and a proportion of invariant rates. The tree improvement was made using the subtree-pruning regrafting (SPR) heuristic search. The reliability of each cluster was determined using the likelihood ratio test (LRT) method as implemented in PhyML. Finally, site-by-site selective pressure was calculated using different models available at the *HyPhy* package. We started by using the simple single likelihood ancestor counting method (SLAC), a counting method that employs maximum likelihood ancestral reconstructions. Then, we applied a more complex dual variable rates model, with the MG94×HKY85 codon rate matrix and dual variable dS and dN rates drawn from bivariate independent discrete distributions, with 4 rate classes.

### Statistical analysis

Statistical analysis was performed in GraphPad Prism version 4.0 for Windows (GraphPad Software), with a level of significance of 5%. Pairwise genetic distances and divergence rates were compared using the Mann Whitney U test.

### GenBank accession numbers

Sequences have been assigned GenBank accession numbers GU230137 - GU230139 (full-length genomic sequences) and EU335962 - EU335903 (C2-C3 sequences).
